# High diagnostic yield of endobronchial ultrasound-guided transbronchial needle aspiration (EBUS-TBNA) in the diagnosis of adolescent pulmonary tuberculosis

**DOI:** 10.1186/s12879-021-06413-z

**Published:** 2021-09-14

**Authors:** Anne Geweniger, Ales Janda, Kristin Eder, Roland Fressle, Cecil Varna Kannan, Hubert Fahnenstich, Mirjam Elze, Christoph Müller, Philipp Henneke, Markus Hufnagel, Roland Elling

**Affiliations:** 1 Department of Pediatrics and Adolescent Medicine, University Medical Center, Medical Faculty, University of Freiburg, Freiburg, Germany; 2 Department of Pediatrics and Adolescent Medicine, University Medical Center Ulm, Ulm, Germany; 3 Practice for Pediatric and Adolescent Medicine, Freiburg, Germany; 4 St. Elisabethen Hospital Lörrach, Lörrach, Germany; 5 Department of Thoracic Surgery, University Medical Center, Medical Faculty, University of Freiburg, Freiburg, Germany; 6 Institute for Immunodeficiency, Center for Chronic Immunodeficiency, University Medical Center, Faculty of Medicine, University of Freiburg, Freiburg, Germany; 7 Berta Ottenstein Program, University Medical Center, Medical Faculty, University of Freiburg, Freiburg, Germany

**Keywords:** Tuberculosis, Diagnostics, Children, Adolescents, Refugee minor, Unaccompanied, Migrants, Refugees, Mediastinal lymphadenopathy, Endobronchial ultrasound-guided transbronchial needle aspiration (EBUS-TBNA)

## Abstract

**Background:**

The microbiological diagnosis of pulmonary tuberculosis (Tb) in a pediatric population is hampered by both low pathogen burden and noncompliance with sputum sampling. Although endobronchial ultrasound-guided transbronchial needle aspiration (EBUS-TBNA) has been found useful for the evaluation of mediastinal pathologies in adults, for children, sparse data are available. Here, we have evaluated EBUS-TBNA as a diagnostic procedure in children and adolescents with suspected pulmonary Tb.

**Methods:**

In this retrospective analysis, we reviewed the charts of unaccompanied refugee minors (URM) who were admitted between January 2016 and July 2018 and who, during their initial medical screening upon arrival in Germany, were found to have abnormal radiological pulmonary and mediastinal findings and/or immunological results indicative of Tb. For each patient, basic sociodemographic data, clinical features and data on diagnostic procedures performed were assessed. These included imaging, immunodiagnostic tests and microbiological data derived from sputum, bronchoalveolar lavage, EBUS-TBNA, bronchoscopy and pleural fluid sampling. All patients who underwent invasive sampling procedures were included in the study.

**Results:**

Out of 42 URM with suspected Tb, 34 fulfilled the study’s inclusion criteria. Ages ranged from 14 to 17 years. All were of African origin, with 70.0% coming from Somalia, Eritrea and Ethiopia. Among the 21 patients for whom EBUS-TBNA was performed, the diagnostic yield was high: 66.7% positive results (MTb detected either by acid-fast stain, culture or PCR in 4.8, 42.9 and 61.9% of samples, respectively). Multidrug-resistant MTb was found in two patients from Somalia. No complications were associated with the procedure. Overall, pulmonary Tb was diagnosed in 29 patients (85.3%), miliary Tb in two patients (5.9%) and latent Tb in three patients (8.8%).

**Conclusions:**

EBUS-TBNA is a sensitive and safe method with high diagnostic yield in the evaluation of pediatric patients with mediastinal pathology and suspected Tb.

**Supplementary Information:**

The online version contains supplementary material available at 10.1186/s12879-021-06413-z.

## Background

The global burden of infections with *M. tuberculosis* (MTb) remains high [1]. Furthermore, in the European Union (EU), MTb infections constitute a substantial public health issue [1]. Although Tb incidence in Germany was at a stable, low level in the years prior to 2013, a significant increase in Tb was seen thereafter, with numbers peaking in 2016 [2]. Largely, this rise was driven by the increase of migrants seeking asylum in Germany during this period. As in many other low-incidence countries, Tb incidence in Germany reflects international migration dynamics: in 2018, 69.8% of newly diagnosed Tb patients in Germany were foreign-born. This translates to an incidence approximately 18 times higher among foreign nationals than among German citizens (37.3 vs. 2.1 cases per 100,000 population, respectively). Patients with a migration background were of younger in age as compared to Germans (age median 28 vs. 59 years). For patients with or without migration background, the pulmonary Tb form prevailed (72.9%) with a low detection rate of multiple drug resistance (3.1%; MDR) [2].

In children, Tb diagnostics are limited both by the lower performance of immunodiagnostic tests, such as the interferon-gamma-release assays (IGRA), and by the low yield of microbiological cultures, due to the paucibacillary nature of the disease [3]. In adolescents, a bacteriological confirmation of pulmonary tuberculosis with positive smear, PCR or culture can be obtained more frequently. However, paucibacillary disease remains a common finding [4]. For several additional reasons, refugees with suspected tuberculosis represent a particularly challenging patient cohort for Tb diagnostics. First, the Tb index patient, (and therefore the drug sensitivity of the infectious MTb), usually is unknown. Second, Tb symptoms, which are often non-specific in children, are even more difficult to elicit due to a variety of factors: language barriers and absence of patient history, as well as the manifestation of symptoms such as weight loss, which may directly result from the poor circumstances of the patient’s migration [5]. Third, empirical Tb treatment based upon mycobacterial resistance patterns in the country of origin is not reliable, because the infection may have occurred in one of the transit countries on the migration route. Fourth, in refugee centers visited by the URMs on their migration route, a significant proportion of patients undergo incomplete treatment [6, 7]. Altogether, these factors underline the utmost importance of culture confirmation of suspected Tb. This should include drug sensitivity testing (DST) for especially vulnerable minor refugees entering the EU.

Among adults, endobronchial ultrasound-guided transbronchial needle aspiration (EBUS-TBNA) has emerged as a valuable procedure for the culture confirmation of suspected MTb intrathoracic lymphadenopathy. This minimally invasive technique allows for ultrasound-guided direct sampling of intrathoracic lymph nodes and it has been shown to increase the sensitivity and culture yield among adult patients with pulmonary Tb [8–11]. However, there is only limited information regarding the utility and safety of EBUS-TBNA in children and adolescents for Tb diagnostics [12–15]. Because many pediatric and adolescent Tb patients initially present with isolated hilar lymphadenopathy and without pulmonary infiltrates [3], sampling techniques such as collection of induced sputum or gastric aspirates typically provide only a low yield of bacterial culture-positivity. The implementation of PCR-based diagnostics have significantly increased the case detection rate of suspected tuberculosis. It has been shown that the pooled sensitivity of GeneXpert in children reaches 62% with a specificity of 92% (with culture as reference standard), and a 82–94% pooled sensitivity in smear positive children aged 0–4 years [16]. However, as culture remains the current diagnostic gold standard, minimal-invasive, direct sampling of enlarged lymph nodes may offer an attractive method for testing pediatric patients with suspected Tb in situations where DST is essential.

Here, we present the results of a retrospective cohort of 34 unaccompanied minor refugees (URM), who, over a two-year period (January 2016–July 2018), were referred to our tertiary care center for workup of suspected pulmonary Tb and who required related invasive diagnostic procedures. Of note, lymph node samples acquired through EBUS-TBNA had the highest yield of all analyzed specimens.

## Methods

### Study population

The study cohort consisted of URM referred to the University Medical Center Freiburg, Center of Pediatrics and Adolescent Medicine, between January 2016 and July 2018, for workup of suspected Tb following infectious diseases screening according to the recommendations of the German Society of Pediatric Infectious Diseases (DGPI), the German Society of Tropical Pediatrics and International Child Health (GTP) and the German Professional Association of Pediatricians (BVKJ) [17]. The patients were retrospectively identified through a standardized review of electronic health records. Inclusion criteria were age ≥ 12 years, a positive IGRA (either enzyme-linked-immuno-spot assay [Tb ELISpot], or QuantiFERON-TB® Gold Plus [QFT]) or tuberculin skin test (TST), plus either clinical symptoms suggestive of Tb (cough > 3 weeks, bloody sputum, weight loss or night sweats) or abnormal radiographic findings. In addition, all patients included underwent an invasive diagnostic workup through bronchoalveolar lavage BAL, along with either pleura fluid sampling and biopsy, bronchoscopy or EBUS-TBNA. EBUS-TBNA was performed when nonivasive sampling methods were negative, and when imaging studies suggested enlarged intrathoracic lymph nodes. The electronic health records review included examination of clinical symptoms at the time of presentation, history of previous Tb treatment and diagnostics during migration, and an inspection of anesthesia protocols for those patients who underwent EBUS-TBNA.

### Diagnostic procedures and imaging

Immunological tests performed included TST and IGRA, by Tb ELISpot or Quantiferon-TB Gold Assay. Imaging included chest X-ray, high-resolution computer tomography scans and magnetic resonance imaging. MR scans or CT scans were performed if chest X-ray findings were inconclusive, especially with respect to the presence of lymphadenopathy, and thereby facilitated the selection of patients suitable for EBUS-TBNA.

Microbiological samples were obtained from sputum, pleural fluid, bronchoalveolar lavage (BAL) and pleural or lymph node biopsy. For all patients admitted, the initial diagnostic procedure was to obtain a sputum sample for microscopy, culture and Xpert– unless they had been transferred from another hospital where sputum diagnostics had been performed already. If patients with lymphadenopathy were negative upon sputum diagnostics, EBUS-TBNA was performed and another sputum sample was obtained following the procedure.

EBUS-TBNA was performed using an Olympus ViziShot system (Olympus Ltd., Tokyo, Japan) equipped with an ultrasonic 7.5 MHz longitudinal transducer. Lymph node biopsies were taken using a 21-gauge needle with 3–5 needle passes for each lymph node.

Microbiological diagnostics included microscopy with Ziehl-Neelsen stain (ZNS), mycobacterial culture and polymerase chain reaction (PCR) (GeneXpert MTB/RIF). Resistance testing was performed genotypically (Hain GenoType MTBDRplus, GeneXpert MTB/RIF) and phenotypically (culture). In addition to microscopy and Ziehl-Neelsen stain, a mycobacterial PCR detection kit (MYCO Direct 1.7 LCD Array kit, Chipron, Berlin) was used as part of histopathological diagnostics in 17 cases.

### Descriptive data analysis

Descriptive data analysis was performed using Microsoft Excel 2019.

## Results

### Sociodemographic characteristics and initial tuberculosis screening results

**Fig. 1:**
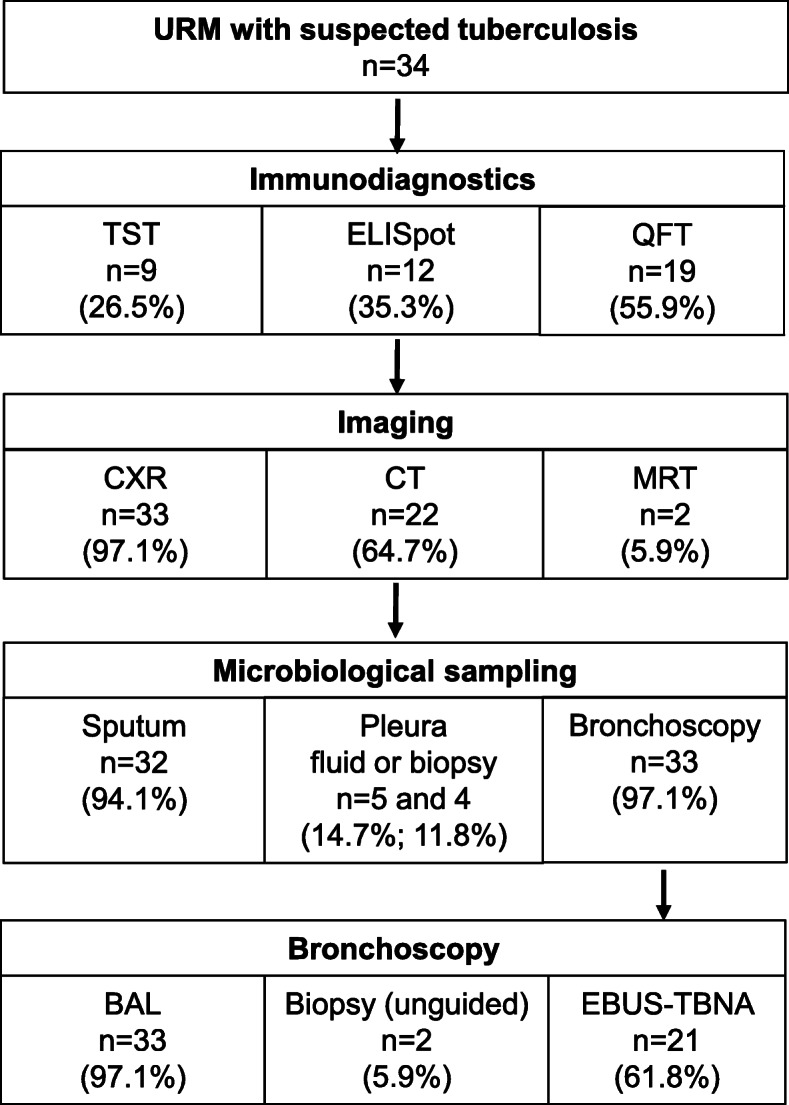
Diagnostic flowchart; TST: Tuberculin skin test, ELISpot: Enzyme-linked-immuno-spot assay, QFT: QuantiFERON-TB® Gold Plus assay, CXR: chest radiograph, CT: computer tomography, MRT: Magnetic resonance tomography, BAL: Brochoalveolar lavage, EBUS-TBNA: Endobronchial ultrasound-guided transbronchial needle aspiration

Among URM patients aged 14–17 (mean 16.0) years, 34 of 42 fulfilled the inclusion criteria. Of these, 33 were male (97.1%) and all were of African origin. East African countries such as Somalia (13/34; 38%), Eritrea (6/34; 18%) and Ethiopia (5/34; 15%) were the top three countries of origin. The most common findings suggestive of Tb were radiological abnormalities in chest X-ray or CT scan (33/34; 97.1%), such as lymphadenopathy, pulmonary infiltrates or pleural effusions, and positive IGRA (28/30; 93.3%). Although TST was positive in all tested patients, it was only performed in nine URM (Table 1). Lymphadenopathy was the most frequent radiological finding (27/34; 79.4%), followed by pulmonary infiltrates (24/34; 70.6%; see table S1). Representative radiographic findings are given in the supplementary Figure 2 and radiological findings of EBUS-positive patients are displayed in supplementary table S3. Six URM (6/34; 17.6%) already had been treated for Tb. No detailed information on the course of prior treatment was available. The diagnostic flowchart is depicted in Fig. 1.

**Table 1:** Initial tuberculosis diagnostics

	n performed	n positive	n negative	%
Chest X-ray or CT-scan abnormalities	34	33	1	97.1
Chest X-ray only	12	12	0	100
Chest CT-scan	22	21	1	95.5
Positive TST	9	9	0	100.0
Positive QFT	19	18	1	94.7
Positive ELISpot	12	10	2	83.3

Each patient tested positive in at least one immunodiagnostic test, except patient Nr. 29, who had a negative QFT. For this patient, further diagnostics were pursued based upon typical radiological findings. In the end, this patient was diagnosed with TB based upon a positive MTb culture from pleural effusion. Three patients (Nr. 18, 22, 25) tested positive in two tests. In two patients (Nr. 5 and 26), the test results were incongruent. Details on clinical and radiologic characteristics of all patients included are displayed in Table S1. Percentage figures in the Table 1 are rounded

*Abbreviations*: *ELISpot* enzyme-linked-immuno-spot assay, *HR-CT* high-resolution computer tomography, *QFT* QuantiFERON-TB® Gold Plus, *MTb M. tuberculosis*, *TST* tuberculin skin test

### Endobronchial ultrasound-guided transbronchial needle aspiration (EBUS-TBNA)

EBUS-TBNA was performed in 21 patients. Table S2 displays the characteristics of procedures performed for these patients. Duration of general anesthesia managed with laryngeal mask airway (LMA) ranged from 25 to 50 min, with an average time of 37.6 min. One patient did not require general anesthesia and received sedation only. Bronchoscopy lasted from 15 to 35 min, with an average duration of 20.7 min. On average, one lymph node was punctured, and five samples obtained. Fourteen of the 21 samples tested positive for MTb. No complications occurred during the procedures.

### Microbiological diagnostics

The microbiological sampling techniques in patients with suspected Tb are described in Fig. 1. Table 2 shows the microbiological test results. A confirmation of MTb was achieved in 73.5% of patients (25/34). PCR diagnostics performed best, with 24 out 34 specimens proving positive (70.6%). Culture-confirmation was achieved in 55.9% (19/34) of patients. Sputum culture, the current gold standard, was positive in only seven out of 32 cases (21.9%). Of all samples obtained, biopsies from lymph nodes or pleura showed the highest sensitivity: 69.6% (16 of 23) and 75.0% (6 of 8).

**Table 2:** Results of microbiological assays (*N* = 34)

Type of sampling	Number of tested patients	Sensitivity of particular MTb detection assay						Aggregate of MTb positivity (per sample type)	
	n	Microscopy^b^		Culture		PCR			
		n	%	n	%	n	%	n	%
Sputum	32	0	0.0%	7	21.9%	6	18.8%	9	28.1%
BAL fluid	33	3	9.1%	8	24.2%	9	27.3%	12	36.4%
LN Biopsy ^a^	23	2	8.7%	9	39.1%	15	65.2%	16	69.6%
Pleural effusion/−biopsy	8	2	25.0%	3	37.5%	4	50.0%	6	75.0%
Aggregate of MTb positivity (per type of assay)	34	5	14.7%	19	55.9%	24	70.6%	25	73.5%

The aggregates in the vertical column represent the diagnostic yield obtained through the respective sample type. The aggregates in the horizontal column represent the diagnostic yield of a particular MTb detection assay across all sample types. Some samples showed positivity in more than one test. This is accounted for in the aggregate numbers. Additionally, in one patient, more than one tissue sample was able to be tested. ^a^21 LN biopsies were obtained by EBUS-TBNA, two by regular bronchoscopy. ^b^Detection of acid-fast bacilli with Ziehl-Neelsen staining.

*Abbreviations BAL* bronchoalveolar lavage, *EBUS-TBNA* endobronchial ultrasound-guided transbronchial needle aspiration, *LN* lymph node, *PCR* polymerase chain reaction, *MTb M. tuberculosis*

Following EBUS-TBNA, a total of 14 patients tested positive for MTb. In these patients, BAL by any method (microscopy, culture, PCR) was positive in 6/14. PCR only from BAL was positive in 3/14 patients, illustrating the additional diagnostic benefit of EBUS-TBNA over BAL alone.

Resistance testing was performed in 32 of 34 cases (94.1%), with two cases receiving culture testing only. Culture and genotypic test results matched in 80% of cases (24 of 30). Of the 25 patients for whom an infection with MTb was microbiologically confirmed, five patients showed mono- or multidrug resistance (MDR). Isoniazid monoresistance was documented in two patients, one from Somalia and one from Eritrea. In one patient from Somalia, streptomycin monoresistance was found. In two patients from Somalia, MDR resistance was discovered. One of the two patients diagnosed with MDR-Tb already had received Tb treatment along the migration route, prior to admission to our hospital.

### Lymph node histology

Histological analysis of lymph node biopsies was available from 21 patients (2/2 non-EBUS guided biopsies, 19/21 EBUS-guided biopsies). From 2/21 patients (9.5%) undergoing EBUS, the material was not sufficient for detailed histological analysis (Table 3). The histological analysis of the lymph node specimens revealed granuloma formation in 14/21 (66.7%) samples. Of note, from the 7 specimens without detectable lymph node granulomas and negative Ziehl-Neelsen staining, a definite diagnosis of tuberculosis could be established through PCR and culture in 6/7 specimens, one sample was only positive by PCR.

**Table 3:** Histopathological findings from lymph node biopsies

Histopathological findings	n	%
Granuloma formation	14/21	66.7
Necrosis	11/21	52.4
Positive Ziehl-Neelsen stain	4/14	28.6
Detection of M.tuberculosis DNA in tissue^a^	9/15	60.0%

^a^kit: MYCO Direct 1.7 LCD Array Kit

### Final diagnosis

Pulmonary Tb was diagnosed in 29 patients (85.4%), miliary Tb in two patients (5.9%) and latent Tb in three patients (8.8%). The most common clinical symptoms were weight loss, chronic cough and dyspnea (44.4, 40.6 and 21.7% respectively; Table S1).

## Discussion

Among children and adolescents, diagnostic yields from microbiologic techniques from bronchoalveolar lavage and sputum provide only a low yield. As a result, many patients have to be treated with empirical anti-tuberculous drugs. EBUS-TBNA is a minimally invasive, diagnostic procedure with relatively high diagnostic yield. Among adults, it is broadly used [18, 19], but in the pediatric population it is rarely applied [15].

The first case of EBUS-TBNA in a 13-year-old male child with sarcoidosis was published in 2009 [20]. To date, four larger, retrospective studies evaluating EBUS-TBNA use in the diagnosis of mediastinal lymphadenopathy, including Tb in children, have been published [12, 14, 21, 22]. Of note, in most of these studies, EBUS-TBNA was not performed for specific Tb diagnostics, but for the workup of enlarged mediastinal lymph nodes of non-infectious origin. Additionally, individual case reports have documented its utility in Tb diagnostics for children and adolescents [13, 23]. Because the use of EBUS-TBNA is limited by the size of airway diameter, the number of children under 12 years old who have been reported to have received EBUS-TBNA is low. In younger patients, an alternative method that does not impede ventilation, e.g., endoscopic ultrasound bronchoscope-guided fine needle aspiration (EUS-B-FNA), needs to be applied [12, 14, 21, 24]. In the literature, the youngest patient described for whom TBNA with a 4 mm scope has been used to evaluate mediastinal lymphadenopathy was a nine-month-old infant [22]. Because the patients in our study were ≥ 14 years of age, there was no anatomical hindrance to using EBUS-TBNA.

Out of the 34 URM eligible for our study, EBUS-TBNA was perfomed in 21 patients. The diagnostic yield of the fine-needle aspiration (14/21; 66.7%) was higher as compared to other pediatric studies (36.0–56.9%) [12, 14, 21, 22], and also as compared to the EBUS-TBNA diagnostic yield reported in adult patients (64.6–96.6%) [19], as well as to meta-analysis with pooled sensitivity of the procedure of 80% [25]. Of the 14/21 patients who were positive through EBUS, culture was positive in 9/14 samples (64.3%), PCR in 13/14 samples (92.9%) and microscopy in 1/14 samples (7.1%). This finding illustrates that at least genomic material of mycobacteria is present in the great majority of enlarged intrathoracic lymph nodes. However, the lower rate of culture positivity compared to PCR detection suggests that lymph node enlargement may persist after successful immunological clearance of replication competent mycobacteria from a lymph node. Of note, our PCR detection rate of 27% from BAL samples was lower than in published pediatric cohorts, reporting a sensitivity of up to 78% [26, 27], We hypothesize this may be due to the mostly paucibacillary disease of our patients, where the majority of pathogen burden is contained within lymph nodes. Overall, EBUS was the only positive diagnostic method via PCR or culture in 7/14 patients, illustrating the benefit of this diagnostic approach.

In our evaluation of several Tb detection methods, the molecular genetic approach using PCR performed best. PCR provided a positivity of 70.6% (24/34), followed by culture proof (19/34; 55.9%) and detection with ZNS (5/34; 14.7%). These findings correspond to already published data for adults [10, 19]. Using a combination of invasive and non-invasive diagnostic approaches allowed us to detect MTb and evaluate drug sensitivity in 93.5% (29/31) active Tb cases. Importantly, a molecular or cultural diagnosis of Tb was possible in 6/7 lymph note specimens without detectable granulomas and a negative Ziehl-Nehlsen staining, illustrating the insufficient negative predictive value of negative histology.

Our detection of drug resistance in 17.2% (5/29) of patients with active Tb (monoresistance in 10.3% (3/29); MDR in 6.9% (2/29) patients) underlines the importance of sample acquisition for drug sensitivity testing. This drug resistance level is higher than has been reported elsewhere (MDR in 3.3.% pediatric and 5.0% adult patients, respectively) [10, 21].

Contrary to study of Gulla et al. [21], where approximately one-fourth of patients were treated with empiric anti-tuberculous medication, none of our patients received any tuberculostatic drugs during the period of microbial sampling. This fact may have contributed to the higher diagnostic yield, especially with respect to culture-positivity.

Six of our patients had a history of tuberculostatic treatment. In two of them, MTb detection failed. For another two, MTb could be detected in sputum (1 x ZNS and PCR; 1 x PCR) and in pleural effusion or BAL (PCR). In one patient, MTb was found in BAL (ZNS, culture, PCR) and in pleural effusion (ZNS and PCR). In the last patient, MTb detection was only possible in a LN sample gained via EBUS-TBNA (PCR).

In our cohort, no complications occurred following EBUS-TBNA. Others have reported rare (i.e., sepsis, pneumothorax, significant hemorrhage) or minor (transient hypoxemia, hypotension or tachycardia, and excessive coughing) complications [10, 12, 14, 21, 28]. EBUS-TBNA is considered to be a safe diagnostic procedure in adults as well as in pediatric patients. Nevertheless, the procedure requires additional training and experience. Due to the low number of pediatric Tb cases pediatric pulmonologists may see, such experience may be difficult to obtain [29]. Our team included both pediatric and adult pneumologists.

### Study limitations

Due to its retrospective design, a significant amount of clinical data was missing. Furthermore, the selected URM cohort we describe was in a particular geographic area in Germany. For these reasons, the epidemiological data cannot be extrapolated to be applicable to all URM in Germany or Europe. In addition, our sample only includes adolescents. This further limits the generalizability of our findings.

Despite its performance, another important limitation of EBUS-TBNA is the requirement of costly endoscopy equipment and adequate training possibilities of pediatric pulmonologists, which is especially limiting in resource-poor settings with a high Tb disease burden.

## Conclusion

Our study demonstrates the utility, safety and feasibility of EBUS-TBNA in a relatively large pediatric population. Its high diagnostic yield may facilitate more accurate diagnostic follow-up for patients with mediastinal lymphadenopathy and suspected Tb. It also may help physicians more clearly identify when tuberculostatic therapy is needed.

## Supplementary Information

**Additional file 1: Table S1.** Clinical and radiologic characteristics of all patients (*n*=34). **Table S2.** Technical details on the EBUS-TBNA procedures (*n*=21). **Table S3.** Radiological findings of patients undergoing EBUS (*n*=14).

**Additional file 2.** Representative radiographic findings.

## Data Availability

The datasets used and/or analysed during the current study are available from the corresponding author on reasonable request.

## Abbreviations

BAL: Bronchoalveolar lavage
BVKJ: German Professional Association of Pediatricians
DST: Drug sensitivity testing
DGPI: German Society of Pediatric Infectious Disease
EBUS-TBNA: Endobronchial ultrasound-guided transbronchial needle aspiration
EUS-B-FNA: Endoscopic ultrasound bronchoscope-guided fine needle aspiration
ELISpot: Enzyme-linked-immuno-spot assay
EU: European Union
GTP: German Society of Tropical Pediatrics and International Child Health
IGRA: Interferon-gamma-release assay
HRCT: High-resolution computer tomography
LN: Lymph node
MRT: Magnetic resonance tomography
MDR: Multi-drug-resistance
MTb: *M. tuberculosis*
QFT: QuantiFERON-TB® Gold Plus assay
PCR: Polymerase chain reaction
Tb: Tuberculosis
TST: Tuberculin skin test
URM: Unaccompanied refugee minor
ZNS: Ziehl-Neelsen stain
