# Supplementary material for: High diagnostic yield of endobronchial ultrasound-guided transbronchial needle aspiration (EBUS-TBNA) in the diagnosis of adolescent pulmonary tuberculosis
Source: BMC Infect Dis. 2021 Sep 14;21:946. doi: 10.1186/s12879-021-06413-z (PMC8439093; doi:10.1186/s12879-021-06413-z)
Supplement: Supplementary file 1 — Additional file 1: Table S1. Clinical and radiologic characteristics of all patients (n=34). Table S2. Technical details on the EBUS-TBNA procedures (n=21). Table S3. Radiological findings of patients undergoing EBUS (n=14). [file 12879_2021_6413_MOESM1_ESM.docx]

**Table S1: Clinical and radiologic characteristics of all patients (n=34).**

|  | n | % |
| --- | --- | --- |
|  |  |  |
| **Radiological chest findings** |  |  |
| Lymphadenopathy | 27/34 | 79.4% |
| Pulmonary infiltrates | 24/34 | 70.6% |
| Pleural effusion | 8/34 | 23.5% |
| Tuberculous cavern | 1/34 | 2.9% |
| Atelectasis | 2/34 | 5.9% |
|  |  |  |
| **Immunoreactivity** |  |  |
| Positive tuberculin skin test | 9/9 | 100% |
| Positive Quantiferon test | 18/19 | 94.7% |
| Positive TB-Spot | 10/12 | 83.3% |
|  |  |  |
| **Signs & symptoms** |  |  |
| Weight loss (≥ 10%) | 8/18 | 44.4% |
| Chronic cough | 13/32 | 40.6% |
| Dyspnea | 5/23 | 21.7% |
| Fever | 4/32 | 12.5% |
| Night sweats | 4/23 | 17.4% |

**Table S2: Technical details on the EBUS-TBNA procedures (n=21).**

| Patient No | Anaesthesia | Bronchoscopy | No. of LN | Number of | Detection of MTb |
| --- | --- | --- | --- | --- | --- |
|  | (Minutes) | (Minutes) | punctured | samples |  |
| 6 | 25 | 15 | 1 | 8 | Yes |
| 10 | 45 | 35 | 1 | 6 | Yes |
| 12 | 45 | 25 | 1 | 5 | Yes |
| 13 | 45 | 25 | 2 | 8 | Yes |
| 14 | 30 | 15 | 1 | 3 | Yes |
| 16 | 30 | 15 | 1 | 5 | Yes |
| 17 | 30 | 20 | 2 | 7 | No |
| 18 | 45 | 25 | 1 | 3 | No |
| 21 | 35 | 15 | 1 | 5 | No |
| 22 | 40 | 20 | 1 | 7 | No |
| 23 | 40 | 20 | 2 | 6 | No |
| 24 | 40 | 15 | 1 | 4 | Yes |
| 25 | 35 | 15 | 1 | 6 | Yes |
| 26 | 0 | 15 | 1 | 6 | Yes |
| 27 | 45 | 20 | 1 | 4 | Yes |
| 29 | 45 | 20 | 3 | 9 | Yes |
| 30 | 50 | 20 | 1 | 4 | Yes |
| 32 | 40 | 25 | 1 | 10 | Yes |
| 34 | 45 | 30 | 1 | 5 | Yes |
| 38 | 40 | 20 | 1 | 3 | No |
| 40 | 40 | 25 | 1 | 4 | No |
| Mean | 37,6 | 20,7 | 1,2 | 5,6 | 14/21 |

*Abbreviations: LN, Lymph node; MTb, Mycobacterium tuberculosis*

**Table S3: Radiological findings of patients undergoing EBUS (n=14).**

|  | n | % |
| --- | --- | --- |
|  |  |  |
| **Radiological chest findings** |  |  |
| Lymphadenopathy | 13/14 | 92.9% |
| hiliary (both sides) | 4/14 | 29% |
| hiliary (either right or left) | 10/14 | 71.4% |
| mediastinal | 2/14 | 14% |
| Pulmonary infiltrates | 9/14 | 64.3% |
| Pleural effusion | 0/14 | 0 |
| Tuberculous cavern | 0/14 | 0 |
| Atelectasis | 0/14 | 0 |
