# Supplementary material for: High diagnostic yield of endobronchial ultrasound-guided transbronchial needle aspiration (EBUS-TBNA) in the diagnosis of adolescent pulmonary tuberculosis
Source: BMC Infect Dis. 2021 Sep 14;21:946. doi: 10.1186/s12879-021-06413-z (PMC8439093; doi:10.1186/s12879-021-06413-z)
Supplement: Supplementary file 2 — Additional file 2. Representative radiographic findings. [file 12879_2021_6413_MOESM2_ESM.pptx]

## Slide 1
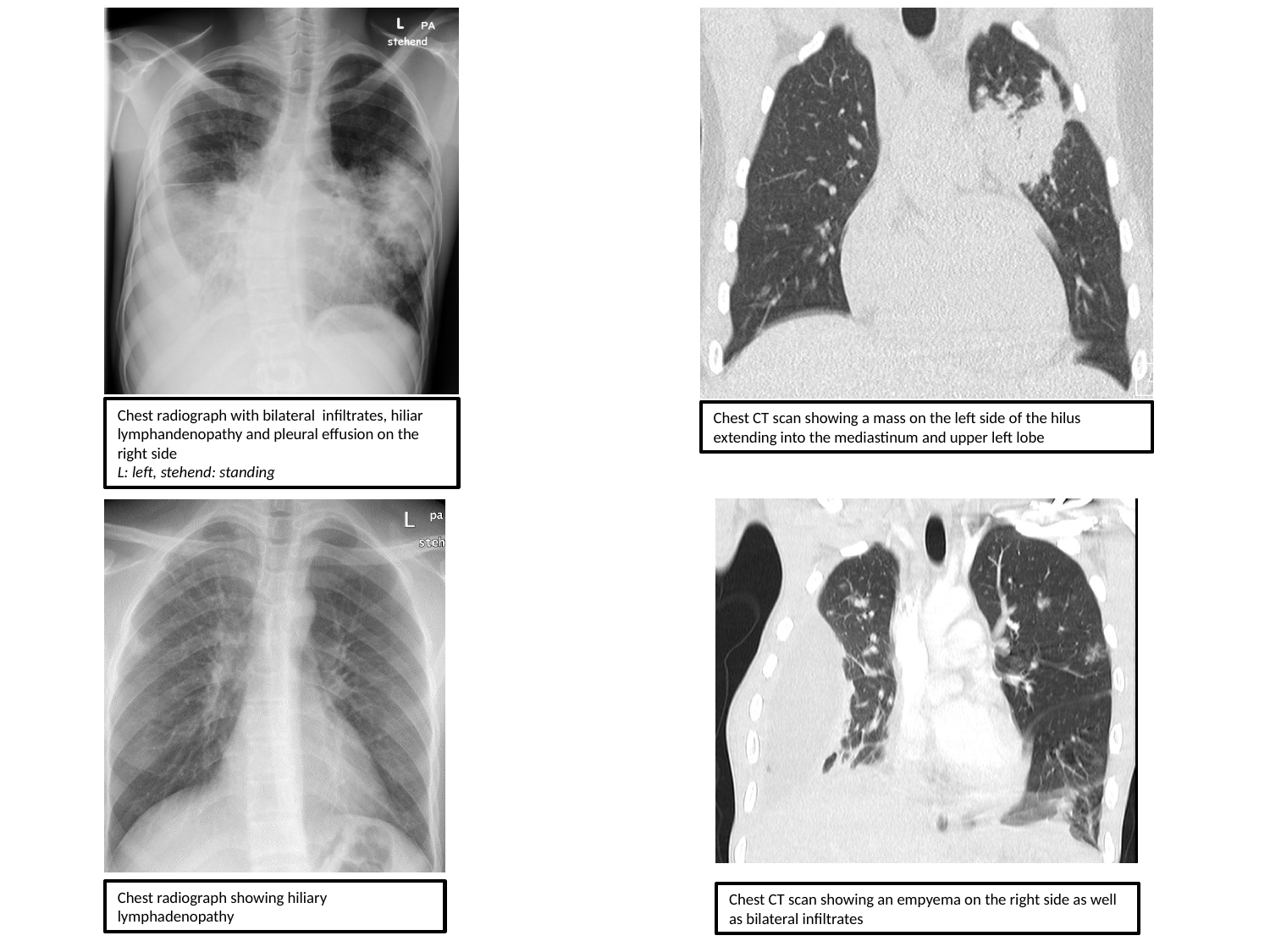

Chest radiograph with bilateral infiltrates, hiliar lymphandenopathy and pleural effusion on the right side
L: left, stehend: standing
Chest CT scan showing a mass on the left side of the hilus extending into the mediastinum and upper left lobe
Chest radiograph showing hiliary lymphadenopathy
Chest CT scan showing an empyema on the right side as well as bilateral infiltrates
